# Ⅱ型冷球蛋白血症的临床特征及预后

**DOI:** 10.3760/cma.j.issn.0253-2727.2021.06.002

**Published:** 2021-06

**Authors:** 虹晓 韩, 欣欣 曹, 薇 苏, 恺妮 沈, 路 张, 道斌 周, 剑 李

**Affiliations:** 1 中国医学科学院、北京协和医学院北京协和医院血液内科 100730 Department of Hematology, Peking Union Medical College Hospital, Chinese Academy of Medical Sciences and Peking Union Medical College, Beijing 100730, China; 2 中国医学科学院、北京协和医学院北京协和医院检验科 100730 Department of Clinical Laboratory, Peking Union Medical College Hospital, Chinese Academy of Medical Sciences and Peking Union Medical College, Beijing 100730, China

**Keywords:** 冷球蛋白, Ⅱ型冷球蛋白血症, 肝炎病毒, Cryoglobulin, Type Ⅱ cryoglobulinemia, Hepatitis virus

## Abstract

**目的:**

探讨Ⅱ型冷球蛋白血症患者的临床特征及预后。

**方法:**

回顾性分析2015年5月至2020年1月北京协和医院确诊的61例Ⅱ型冷球蛋白血症患者的临床资料。

**结果:**

61例患者中，男性26例（42.6％），中位诊断年龄为53（28～79）岁。继发病因包括丙型肝炎病毒（HCV）感染（21.3％）、乙型肝炎病毒（HBV）感染（21.3％）、自身免疫性疾病（14.8％）和血液系统肿瘤（11.5％）。31.1％患者为特发性。常见首诊症状包括皮肤紫癜、蛋白尿、血尿、肾功能不全、发热及关节痛。实验室检查显示，中位冷球蛋白水平为215.9（22.0～17 075.8）g/L，54例（88.5％）为IgM单克隆。类风湿因子（RF）升高患者占93.2％，C3下降患者占57.6％，C4下降患者占61.0％。共49例（80.3％）患者接受治疗，总体临床缓解率为75.5％，预计3年总生存率为89.3％。

**结论:**

Ⅱ型冷球蛋白血症是一种多系统受累的全身性疾病，病因以肝炎病毒感染多见。早期诊断和干预对于改善预后有重要意义。

## Abstract (en)

**Objective:**

To explore the clinical characteristics and outcome of patients with type Ⅱ cryoglobulinemia in our center.

**Methods:**

Clinical data of 61 patients diagnosed with type Ⅱ cryoglobulinemia in Peking Union Medical College hospital from May 2015 to January 2020 were retrospectively analyzed.

**Results:**

A total of 61 patients were enrolled in the study, including 26（42.6％） males, with a median age of 53（range 28–79）years. The primary diseases identified were hepatitis C virus infection（21.3％）, hepatitis B virus infection（21.3％）, autoimmune diseases（14.8％）, and hematological tumors（11.5％）. Idiopathic cryoglobulinemia patients accounted for 19 cases（31.1％）. The major symptoms presented were purpura, proteinuria, hematuria, renal failure, fever and arthralgia. The median concentration of cryoglobulin level was 215.9（22.0–17 075.8）g/L, and the IgM-monoclonal component of cryoglobulin was identified in 54 patients（88.5％）. Rheumatoid factor increased in 93.2％ of patients. C3 decreased in 57.6％ of patients. C4 decreased in 61.0％ of patients. Treatment was initiated in 49 patients（80.3％）, and the total clinical remission rate was 75.5％. The expected 3-year overall survival was 89.3％.

**Conclusion:**

Type Ⅱ cryoglobulinemia was a systemic disease with multi-organ involvement. Most type Ⅱ CGs were secondary to hepatitis virus infection. Early diagnosis and proper treatment could bring better outcome.

冷球蛋白（cryoglobulin）是一种低温下会出现沉淀、复温至37 °C后又能重新溶解的免疫球蛋白。1974年Brouet等[1]根据冷球蛋白的组成将冷球蛋白血症分为3型：Ⅰ型由单纯的单克隆免疫球蛋白组成；Ⅱ型由单克隆免疫球蛋白与多克隆免疫球蛋白组成；Ⅲ型由多克隆IgM和多克隆IgG组成。由于该病的发病率较低，我国能够开展冷球蛋白检测的中心较少，因此冷球蛋白血症的漏诊、误诊率较高。本研究回顾性分析了北京协和医院61例Ⅱ型冷球蛋白血症患者的临床表现、治疗和转归，以提高临床医师对此病的认识。

## 病例与方法

1. 病例：回顾性分析2015年5月至2020年1月北京协和医院确诊的61例Ⅱ型冷球蛋白血症患者的人口学资料、实验室检查及治疗方案。

2. 冷球蛋白测定：冷球蛋白的检测过程与既往文献报道一致，使用提前预热的试管抽取静脉血，在37 °C恒温条件下送检[2]。孵育离心出血清后，将其在4 °C条件下保存7 d，观察沉淀情况。如7 d内血清出现沉淀，则将其重新复温至37 °C。如沉淀重新溶解，则冷球蛋白定性为阳性。将冷沉淀纯化后通过免疫电泳按照Brouet标准对冷球蛋白进行分型[1]。采用文氏管测定冷沉淀比容、直接测定冷沉淀中冷球蛋白含量两种测定方法进行冷球蛋白定量[3]。

3. 受累脏器：参照文献[4]，脏器受累定义如下：①皮肤：由临床医师诊断的紫癜样皮疹，皮肤溃疡或坏死，网状青斑或寒冷相关的荨麻疹、紫绀及雷诺现象等；②周围神经：经神经专科医师诊断和（或）肌电图证实的周围神经病；③肌肉及关节：存在肌痛和关节肿痛等症状；④肾脏：出现肾功能不全（血清肌酐定量>132 µmol/L）和（或）24 h尿蛋白>0.5 g和（或）尿潜血阳性，且能除外其他肾脏疾病，或经肾脏活检病理证实为冷球蛋白相关肾小球肾炎；⑤心脏：冠状动脉病变或心肌病变，且能除外其他心脏疾病[5]；⑥肺脏：闭塞性细支气管炎、肺间质病变、弥漫性肺泡出血。

4. 继发性病因：继发性病因包括以下四类。（1）肝炎病毒感染：①丙型肝炎病毒（HCV）感染：抗HCV抗体阳性或HCV-RNA大于10^3^拷贝/ml[6]；②乙型肝炎病毒（HBV）感染：HBV表面抗原（HBsAg）阳性或HBV-DNA大于10^3^拷贝/ml或在冷球蛋白中分离出HBsAg。（2）血液系统肿瘤：包括慢性淋巴细胞白血病/小淋巴细胞淋巴瘤（CLL/SLL）、华氏巨球蛋白血症（WM）和多发性骨髓瘤（MM）[7]。（3）自身免疫性疾病：包括系统性红斑狼疮、干燥综合征、复发性多软骨炎、未分化结缔组织病等[8]。（4）特发性：未发现继发性病因的冷球蛋白血症。

5. 治疗：①单纯抗肝炎病毒治疗；②以糖皮质激素为主的治疗：糖皮质激素±免疫抑制剂±抗病毒治疗、糖皮质激素+苯丁酸氮芥治疗；③以利妥昔单抗为基础的化疗：利妥昔单抗单药、DRC方案（地塞米松+利妥昔单抗+环磷酰胺）、R-CVP方案（利妥昔单抗+环磷酰胺+长春新碱+泼尼松）；④以硼替佐米为基础的化疗：BCD方案（硼替佐米+环磷酰胺+地塞米松）；⑤支持治疗：保暖等。

6. 疗效评估：参照文献[9]–[10]进行疗效评估，包括临床症状评估及血液学评估。临床症状评估包括①临床完全缓解（CR）：定义为疾病活动的所有临床表现消失；②临床部分缓解（PR）：指半数及以上临床症状改善；③临床无缓解（NR）：症状无改善。血液学评估包括①血液学CR：定义为冷球蛋白转阴、类风湿因子（RF）正常、C3及C4恢复正常；②血液学PR定义为冷球蛋白定量较基线下降50％及以上、RF活性较基线下降50％及以上、C3及C4较基线升高50％及以上；③血液学NR定义为冷球蛋白、RF、C3及C4定量变化未达PR标准。

7. 随访：通过门诊或电话进行随访，末次随访时间为2020年9月1日。总生存（OS）时间定义为自确诊至患者死亡或末次随访的时间间隔。

## 结果

1. 临床资料：61例患者中，男性26例（42.6％），女性35例（57.4％），诊断时中位年龄53（28～79）岁。26例（42.6％）患者继发于肝炎病毒感染，其中HCV及HBV感染各13例（21.3％）。9例（14.8％）患者继发于自身免疫性疾病，其中系统性红斑狼疮6例（9.8％），干燥综合征、复发性多软骨炎和未分化结缔组织病各1例（1.6％）；血液系统肿瘤7例（11.5％），其中不能分型的B细胞淋巴增殖性疾病4例（6.6％），CLL、MM、WM各1例（1.6％）。另有19例（31.1％）为特发性。

2. 临床表现及受累器官：57例（93.4％）患者具有各种临床症状。皮肤受累者37例（60.7％），以皮肤紫癜最为常见（32例）。雷诺现象3例（4.9％）。肾脏受累32例（52.5％），其中蛋白尿32例（52.5％）、血尿23例（37.7％）、肾功能不全22例（36.1％）。32例肾脏受累患者中位血清肌酐149（51～370）µmol/L，中位24 h尿蛋白为3.19（0.36～13.61）g。在18例行肾活检的患者中，毛细血管内增生性肾小球肾炎9例，膜增生性肾小球肾炎8例，结节硬化性肾小球肾炎1例。关节受累者14例（23.0％），表现为关节痛（13例）和关节肿胀（5例）。周围神经受累者10例（16.4％），均表现为感觉神经病变。肺受累者3例（4.9％），表现为呼吸困难，胸部高分辨CT均提示肺间质改变。心脏受累者5例（8.2％），均表现为心肌病变和急性心力衰竭。

3. 实验室检查：文氏管测定冷沉淀比容法测定的中位冷球蛋白定量为2％（0～90％），其中1.0％～5.0％、5.1％～20.0％及>20.0％者分别为30例（48.4％）、9例（14.5％）和2例（3.2％）。直接测定冷沉淀中冷球蛋白定量的中位数为215.9（22.0～17 075.8）g/L。在单克隆免疫球蛋白类型中，IgM型冷球蛋白共54例（88.5％），其中IgM κ型46例（75.4％），IgM λ型8例（13.1％）；IgG κ型2例（3.3％），IgG λ型、IgA κ型和IgA λ型各1例（1.6％）。2例患者因冷沉淀为凝胶状，单克隆成分无法分型。RF升高者占93.2％（41/44），中位RF水平为350（1～52 172）IU/ml。C3降低者占57.6％（34/59），C4降低者占61.0％（36/59）。

4. 治疗方案与临床结局：接受治疗的49例患者的治疗方案和疗效见图1。24例肝炎病毒感染的患者均给予了抗病毒治疗：在HCV感染者中，1例心脏受累者接受了利妥昔单抗治疗，3例肾脏受累者接受了激素±环磷酰胺治疗；在HBV感染者中，1例心脏受累及1例肾脏、皮肤、关节多系统受累者接受了利妥昔单抗治疗，6例肾脏受累者接受了激素±环磷酰胺治疗。9例自身免疫性疾病患者接受了激素±环磷酰胺。7例血液系统肿瘤患者均基于其基础病进行了化疗。19例特发性患者中，5例接受了激素+环磷酰胺治疗，3例分别接受利妥昔单抗单药、DRC方案和R-CVP方案治疗，1例接受BCD方案治疗，9例无症状和（或）仅有皮肤受累者未治疗，1例失访。

**图1:**
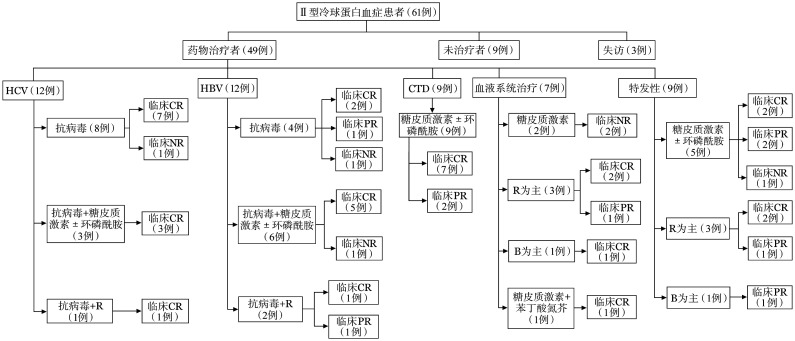
61例Ⅱ型冷球蛋白血症患者的治疗与疗效 HCV：丙型病毒肝炎；HBV：乙型病毒肝炎；CTD：结缔组织病；R：利妥昔单抗；B：硼替佐米；CR：完全缓解；PR：部分缓解；NR：无缓解

在可行血液学疗效评估的35例患者中，达CR和PR者分别有15例（42.9％）和16例（45.7％）。在49例可行临床疗效评估的患者中，CR率为75.5％（37/49）。在肾脏受累的32例患者中，获得临床CR、PR和NR的患者分别有22例（68.8％）、7例（21.9％）和3例（9.4％）。5例心脏受累的患者中，获得临床CR者4例（80％），1例失访。3例肺脏受累者中，获得临床CR和NR者分别有2例（66.7％）和1例（33.3％）。

中位随访20（2～63.5）个月，死亡7例，3例死于原发病进展，3例死于治疗相关感染，1例死于肺栓塞。1年和预期3年OS率分别为93.4％和89.3％（图2）。

**图2:**
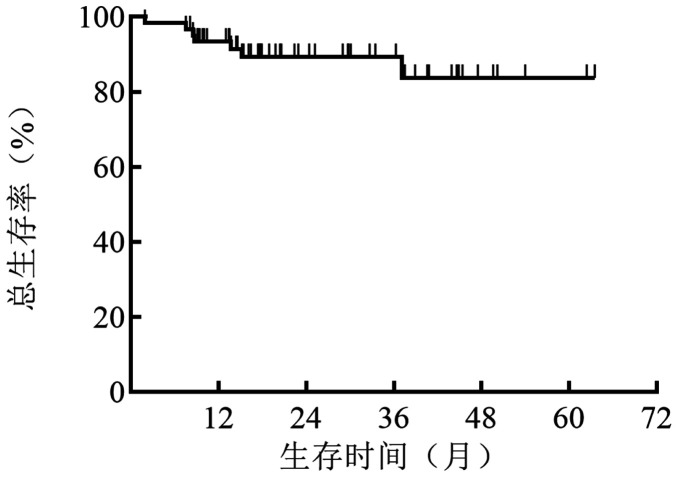
61例Ⅱ型冷球蛋白血症患者的总生存曲线

## 讨论

冷球蛋白血症是一种少见疾病，本研究依据Brouet分类纳入了61例初治Ⅱ型冷球蛋白血症患者进行临床分析。Ⅱ型冷球蛋白血症常继发于病毒感染，以HCV感染最为常见，其他还包括HBV及人类免疫缺陷病毒（HIV）[10]–[11]。非感染性病因主要包括结缔组织病和血液系统肿瘤。2012年，一项回顾性研究纳入242例非感染相关Ⅱ型冷球蛋白血症患者，30％的患者继发于免疫系统疾病，22％的患者继发于血液系统肿瘤，48％为特发性[12]。在本研究中，HBV感染者与HCV感染者相当，可能与我国HBV感染率高有关。

Ⅱ型冷球蛋白血症的临床表现具有高度异质性。免疫复合物介导的血管炎是该病导致不同临床表现及器官损害的主要机制[13]。冷球蛋白血症患者出现临床症状的比例为2％～50％，皮肤是最常见的受累部位，超过90％的皮肤受累患者有皮肤紫癜表现[14]。既往文献报道，肾脏和周围神经受累比例约为30％，心脏、肺脏、中枢神经系统受累比例为1％～5％[10]。本研究中，6.6％的患者为无症状患者，皮肤同样是最常见的受累部位，其次为肾脏和周围神经。

Ⅱ型冷球蛋白血症中的冷球蛋白定量多小于5％[15]。Ⅱ型冷球蛋白由具有RF活性的单克隆球蛋白与多克隆球蛋白构成，因此，Ⅱ型冷球蛋白血症患者的RF活性明显升高。由于免疫复合物的形成导致补体系统经典途径的激活和消耗，补体水平多减低，且以C4减低更为明显[16]。在本组患者中，81％患者冷球蛋白水平小于5％。对于一些无法检测冷球蛋白的中心，临床疑诊该病时，高水平的RF活性及低水平补体有一定的诊断参考价值。

Ⅱ型冷球蛋白血症目前尚无标准的治疗方案，治疗原则是针对原发疾病的病因治疗及免疫抑制治疗。对于感染相关Ⅱ型冷球蛋白血症患者[17]，有肝炎病毒活动证据者需予抗病毒治疗，继发于血液肿瘤患者应积极治疗原发血液肿瘤。对于仅有皮肤紫癜、关节痛、周围神经病变的轻中度患者，可单纯治疗原发病或在其基础上加用小剂量激素；有严重血管炎表现者，如严重肾功能不全、胃肠道受累等，需同时接受大剂量激素和（或）环磷酰胺治疗，还可考虑利妥昔单抗治疗；对于进展迅速者，如急进性肾小球肾炎，心脏、肺脏、中枢神经系统受累等患者，在上述治疗的基础上还需考虑血浆置换[18]–[19]。

国外文献报道，在Ⅱ型冷球蛋白血症患者中，HCV感染相关者的10年OS率为75％[20]，非感染相关者1年、5年的OS率分别为91％、79％，死亡的主要原因为治疗相关感染（50％）[21]。本研究截至随访终点共7例患者死亡，其中4例死于治疗相关感染。这提示我们在治疗过程中应高度警惕感染发生。

综上，当患者有皮肤、肾脏、周围神经等多系统受累症状伴RF升高或补体降低时，需考虑冷球蛋白血症可能。治疗主要基于治疗原发病及免疫抑制治疗，患者总体预后良好。
